# The Institutional Worker

**Published:** 1920-01-10

**Authors:** 


THE
### INSTITUTIONAL WORKER
**Being a Special Supplement to "The Hospital"**

### OUR BUREAU OF INFORMATION.

### Rules for Correspondents.

1. Every letter must be accompanied by the coupon to be cut from the back cover (inside page) of The Hospital, current issue, and must contain the name and address of the correspondent with pseudonym for publication if desired. Replies by post cannot be given save under exceptional circumstances at the Editor's discretion.
2. Letters from Approved Homes in reply to special needs published in the Bureau should state terms and full particulars, and be sent prepaid under cover to the Editor of the Bureau with name written across coupon for identification.
3. Proprietors of Homes which have not yet been entered on the List of Approved Homes, but have spare accommodation likely to suit special needs, are invited to write for an application form for registration. The fee for registration, which includes two announcements of the Home in the Bureau and other privileges, is 10s.
4. All communications to be addressed to the Editor of The Hospital, 28 Southampton Street, Strand, London, W.C. 2, and marked "Bureau of Information."

### INSTITUTION FACTS AND FIGURES.

### Obstacles to Welfare Work.

#### CONDITIONS IN DURHAM.

Now that maternity and child-welfare schemes have been in actual practice for some time, the authorities are beginning to find out weak points. Thus Dr. Mabel Brodie, reporting to the Durham County Council, declares that before the work in the county can be of definite value a great deal more ante-natal work will have to be undertaken. This is always the most difficult part of the work to develop, partly because of the reluctance of the women to come to the centre unless the doctor has fully gained their confidence, and partly because of lack of support from the doctor and the midwife, until they too realise that ante-natal clinics feed them instead of withdrawing work from them. Home visiting, too, is essential, and many cases can be sent by the health visitor, who sees the expectant mother in her own home.

Several expectant mothers are now coming to the centres in the county, but the work is hampered through (1) lack of the necessary equipment, (2) lack of suitable accommodation, (3) midwives do not seem to send up many cases, though one or two have done so. Probably the district nurse midwives, when they are fully established, will be of considerable help in this branch of the work. (4) Lack of a good hot-water supply, which is essential when doing ante-natal work. (5) Necessary privacy.

The provision of a suitable special consultative centre or centres to which cases can be sent adds greatly to the usefulness of the work. These will offer themselves, Dr. Brodie anticipates, when the maternity hospitals open under the county; meantime there is only the Maternity Hospital, Newcastle, to which Durham cases can be sent where the accommodation is very limited. Dr. Brodie is strong on the point that more ante-natal work would tend to increase the amount of breast-feeding in the county. It would be possible to have a good ante-natal clinic in Durham itself, but a day apart from that on which the child-welfare centre is held would have to be arranged. This would not be practicable at the other centres at present, but a definite hour could be fixed for seeing ante-natal cases.

Dr. Brodie deals comprehensively with artificial feeding, which seems to be resorted to for any cause whatever. In this connection the report states :—

"The mother's usual tale is "the milk left me," and this may be even at the fourteenth day, when a good milk supply is not yet established and when perseverance and massage will do much towards increasing the quantity and improving the quality of the milk supply. The difficulty is to get into touch with the mothers so early as the fourteenth day. This must be in the hands of the doctor, midwife, and health visitor, all of whom have access to the mother during the puerperium. They can emphasise the necessity of perseverance and see that massage and sponging are carried out. In practically no case has this been done before they came to the centre. Mothers have persevered and have re-established a failing milk supply to their own satisfaction and that of their baby. Advice at the centre should be followed up by one or two subsequent visits by a health visitor, who can see that the doctor's orders are carried out. Massage could be done by the County Nursing Association's nurses when they are in full working order.

"To keep in close touch with the infant and mother an adequate health visitor staff is absolutely essential, else the work can only be attempted and not done. With the present staff very little revisiting is possible and intimate co-operation with the work of the centres is lacking. The ideal is to have a centre in each health visitor's district.

"The main object of many mothers in coming to the centre is "Glaxo" and not advice, though many are very glad to see the doctor and the nurse. The mothers require more education in the feeding of their infants. Thanks to the advice of the health visitors, the medical officers and the efforts of the voluntary workers, the majority of the infants are clothed satisfactorily. Any defect in clothing is quickly remedied at the centres, where advice is given and where model clothes or patterns can be obtained from the health visitors and the voluntary workers, where we are fortunate enough to have the latter; and there is no doubt of the value of a reliable voluntary worker."

#### DANGERS OF X-RAY ROOMS.

The fatality recently reported as occurring in an *x*-ray room, when a French radiographer was electrocuted while examining a patient, need not alarm you. Provided you take certain precautions, which are based on fundamental truths, there can be no possible danger of your suffering in the same way. In the case reported, a high-tension lead from the transformer to the tube was in contact with the metal tube stand, which was not earthed; consequently, when the doctor grasped the stand to adjust the tube his body formed a conduction from the transformer to earth. Doubtless both hands were on the stand, and he could not release them to switch off the current. All high-tension leads should be kept taut; if a lead sags over at its connection with the tube it is a source of danger. A properly acting spring tape will effect this. Metal parts of apparatus should be earthed. Although this may cause undue strain on the installation of certain types of *x*-ray coils, if a high-tension lead comes in contact with it this risk is the lesser of two evils. It is quite unnecessary to adjust a tube stand whilst the current is passing through the tube. It is so simple first to switch off the current; the risk in any case is one which few radiographers would take. Resistance boards should be fitted with the requisite safety fuses. These are often conspicuous by their absence. Trolly wires conveying the high-tension current should be carefully examined periodically. It is, of course, possible to provide for their unavoidable earthing should they break whilst alive, but the requisite earthed wires crossing beneath them are likely to be a source of inconvenience.—K. W. S.

### Question for January.

### UTILISING VOLUNTARY HELP.

How would you deal with offers of voluntary part-time help in connection with your hospital from professional men, clerks, &c.? Give suggestions how their assistanee might be utilised.

**(A minimum payment of 10s. 6d. will be made for each published answer.)**

### RULES.

The following rules must be observed:—

1. Contributions must be written on one side of the paper. Conciseness and terseness are desirable features. The MS. must bear the name and address of the sender and be accompanied by coupon to be cut from the back cover (inside page) of the current issue of THE HOSPITAL. A pseudonym must be chosen if the name is not to be published.

2. Contributions must be addressed to the Editor of THE HOSPITAL Institutional Worker Supplement, 28 & 29 Southampton Street, Strand, London, W.C. 2, should reach him before the end of the current month, and be marked in the left-hand corner "Facts and Figures."

### QUESTIONS INVITED.

IN connection with our Question Box, we offer every month five shillings each for the best questions which are sent in for consideration. The questions may be on any institutional subject and concern any department, from the secretary's to the porter's, or the matron's to the domestic staff; the one essential is that they must be practical and deal with points that have a definite relation to hospital or institutional administration, upkeep, finance, or management. Points relating to artificers' work, housekeeping, the laundry, out-patients, and other practical matters will be welcome. It is hoped that thus, when difficult or doubtful points arise in the course of their work, institutional workers will be encouraged to put them in the form of a question and send them to the Editor, THE HOSPITAL Bureau of Information, 28 & 29 Southampton Street, Strand, London, W.C. 2, marked "Question Box."

The best questions will be published in due course and our readers will be given the opportunity of answering them. Thus all institutional workers may be able to co-operate with the view of helping the work and smoothing the difficulties of each other. In short, we want the Question Box of the *Institutional Worker* to be freely used by every worker habitually.

### ENQUIRIES AND ANSWERS.

### *SICK AND IN NEED.*

### Convalescent Home in Scarborough.

THE Yorkshire Convalescent Home and House of Rest for Ladies, St. Martin's Lodge, South Cliff, Scarborough, is open during the winter, with the exception of the month of February. Admission in the winter months is by payment of 15s. per week; medical attendance is free. If 15s. is more than you can afford per week, the Home has several free fortnights (we presume by letters), and if you write to the Hon. Secretary and explain your circumstances you will probably be fortunate in receiving one.—MRS. K.

### *EMPLOYMENT AND TRAINING.*

### Post of Seamstress.

WE do not know of any hospitals requiring a seamstress at the present time. There are very few hospitals that do not employ one, and it is open to you to write to the matrons of any institution expressing your need. You will find a list of hospitals in "Burdett's Hospitals and Charities," a copy of which you will find in any public library. The only other alternative is to watch the advertisements which appear in the nursing and daily journals. A coupon should be enclosed with each inquiry. We regret we cannot give postal replies.—E. D.

### Poor-Law Training and Private Nursing.

WE know of many nurses who have been trained in some of the well-known Poor-Law infirmaries who are holding good positions in hospitals and who do well in private nursing. You must remember that the personality of the nurse counts for much, and, other things being equal, this will carry her far.—M. L.

### Part-time Massage Post.

WE suggest that you write to the County Director, British Red Cross Society, 24 Eaton Square, S.W. 1, with a view to obtaining a part-time post in one of the orthopædic clinics under their administration. It is essential that you should hold the electrical certificate of the I.S.T.M., as well as that for massage.—MABELLE.

### Draft Regulations for the Training of Midwives.

YOU can obtain the prefatory memorandum of the Draft Regulations for the Training of Midwives (Board of Education), and the circular issued by the Ministry of Health dealing with the Maternity and Child-welfare Regulations, upon application to His Majesty's Stationery Office, Imperial House, Kingsway, W.C. 2, 1d. net. You must be approved by the Central Midwives Board for the purpose of teaching midwifery.—JANET.

### *MISCELLANEOUS.*

### Dressing and Bedside Stands.

WE do not advocate glass shelves for dressing stands. It is true that they look attractive, but they are a source of continual expense, and especially so in frosty weather. Enamelled metal is quite as aseptic, much cheaper, and more lasting. Messrs. Whitfield, Ltd., of Birmingham, are makers of excellent dressing stands. They make also a very good bedside stand with three shelves, dished to prevent articles from rolling off, and a lattice-work cupboard fitted with lock and key. The lattice-work makes it possible for the nurse to see what is kept by the patient, and uncleanliness is therefore readily detected.—C. A. S.

### Dinner-Wagons.

WE do not advocate large dinner-wagons capable of supplying the whole of the wards; they are cumbersome and noisy at the best, and, moreover, the ward last served gets the food unduly cooled at the end of a long round. We suggest a small wagon for each ward or floor. We fear that you will get nothing suitable at the present time under £10 or £12, unless, of course, you have a carpenter who could make you one. Messrs. J. Christopher and Sons, Ltd., of 39 Clerkenwell Road, London, are a reliable firm, and make a speciality of this kind of work.—"IMPROVEMENT."

### *THE HOUSEKEEPERS' DEPARTMENT.*

### An Excellent Fruit Cake.

FOR a good-sized cake you will require 1½ lb. of margarine, 2½ lb. currants, ¾ lb. of mixed peel, ¼ or ½ lb. blanched almonds, eight dried or new-laid eggs, 2½ lb. flour, and 1½ lb sugar. Mix in the usual way, and bake in a moderate oven for six hours. It is a good plan to place the tin on a plate of salt; this prevents the bottom of the cake burning and becoming hard.—"EXTRAVAGANT ONE."

### Egg Flip.

YOU will require half an ounce of powdered sugar to the yolks of two eggs; eight tablespoonfuls of brandy, and the same number tablespoonfuls of cinnamon water. Beat the sugar and yolks of eggs together; mix the brandy and cinnamon water together; then mix and beat the whole ingredients thoroughly.—PRIVATE NURSE.

### Using-up Jellies.

IT is quite a good plan to use up jellies in the following way: Break up the jelly into a basin, whisk well with an egg whisk into the appearance of a "froth," place in a small glass dish, and cover with either white of egg or whipped cream. This makes a dainty sweet for an invalid.—W. S. T.

